# Induced Differentiation of Human Myeloid Leukemia Cells into M2 Macrophages by Combined Treatment with Retinoic Acid and 1α,25-Dihydroxyvitamin D_3_

**DOI:** 10.1371/journal.pone.0113722

**Published:** 2014-11-19

**Authors:** Hiromichi Takahashi, Yoshihiro Hatta, Noriyoshi Iriyama, Yuichiro Hasegawa, Hikaru Uchida, Masaru Nakagawa, Makoto Makishima, Jin Takeuchi, Masami Takei

**Affiliations:** 1 Division of Biochemistry, Department of Biomedical Sciences, Nihon University School of Medicine, Tokyo, Japan; 2 Division of Hematology and Rheumatology, Department of Medicine, Nihon University School of Medicine, Tokyo, Japan; Beth Israel Deaconess Medical Center, Harvard Medical School, United States of America

## Abstract

Retinoids and 1α,25-dihydroxyvitamin D_3_ (1,25(OH)_2_D_3_) induce differentiation of myeloid leukemia cells into granulocyte and macrophage lineages, respectively. All-*trans* retinoic acid (ATRA), which is effective in the treatment of acute promyelocytic leukemia, can induce differentiation of other types of myeloid leukemia cells, and combined treatment with retinoid and 1,25(OH)_2_D_3_ effectively enhances the differentiation of leukemia cells into macrophage-like cells. Recent work has classified macrophages into M1 and M2 types. In this study, we investigated the effect of combined treatment with retinoid and 1,25(OH)_2_D_3_ on differentiation of myeloid leukemia THP-1 and HL60 cells. 9-*cis* Retinoic acid (9cRA) plus 1,25(OH)_2_D_3_ inhibited proliferation of THP-1 and HL60 cells and increased myeloid differentiation markers including nitroblue tetrazolium reducing activity and expression of CD14 and CD11b. ATRA and the synthetic retinoic acid receptor agonist Am80 exhibited similar effects in combination with 1,25(OH)_2_D_3_ but less effectively than 9cRA, while the retinoid X receptor agonist HX630 was not effective. 9cRA plus 1,25(OH)_2_D_3_ effectively increased expression of M2 macrophage marker genes, such as *CD163*, *ARG1* and *IL10*, increased surface CD163 expression, and induced interleukin-10 secretion in myeloid leukemia cells, while 9cRA alone had weaker effects on these phenotypes and 1,25(OH)_2_D_3_ was not effective. Taken together, our results demonstrate selective induction of M2 macrophage markers in human myeloid leukemia cells by combined treatment with 9cRA and 1,25(OH)_2_D_3_.

## Introduction

Retinoids play roles in numerous biological functions, such as cellular proliferation and differentiation, embryogenesis, immunity and metabolism [1]. An active natural retinoid, all-*trans* retinoic acid (ATRA), is effective in differentiation therapy for acute promyelocytic leukemia (APL) [2]. APL is a subtype of acute myeloid leukemia, which is characterized by a specific chromosomal abnormality t(15,17) associated with a genetic rearrangement between retinoic acid receptor α (RARα) (gene symbol, *RARA*) and the promyelocytic leukemia gene *PML* [2]. RARα plays a role in granulocytic differentiation of hematopoietic cells and the abnormal chimeric receptor PML-RARα has been implicated in APL pathogenesis by blocking the myeloid differentiation program and enhancing self-renewal of leukemic cells [3], [4]. Pharmacological doses of ATRA induce differentiation of APL cells into granulocytes through degradation of PML-RARα and recovery of physiological RARα signaling [3], .

Retinoids, including ATRA, 9-*cis* retinoic acid (9cRA) and synthetic RAR ligands, exhibit anti-tumor effects not only on APL but also on other malignancies, such as breast cancer, lung cancer, and head and neck cancer [5]. With regard to leukemia, ATRA was first reported to induce the differentiation of human myeloid leukemia HL60 cells towards the granulocytic lineage [6], [7]. Importantly, HL60 cells are derived from non-APL leukemia without t(15,17) [8], and ATRA can also induce differentiation of leukemia cells from non-APL myeloid leukemia patients [9]. Retinoids in combination with other differentiation inducers, such as 1α,25-dihydroxyvitamin D_3_ (1,25(OH)_2_D_3_) and dibutyryl cAMP, synergistically induce differentiation of leukemia cells [10]–[13]. However, the underlying mechanisms of retinoid-induced differentiation of leukemia cells remain poorly understood and retinoids have not been utilized in the treatment of myeloid leukemia other than APL.

The active form of vitamin D_3_, 1,25(OH)_2_D_3_, regulates calcium and bone homeostasis, immunity, and cellular growth and differentiation through direct binding to the vitamin D receptor (VDR), and has been demonstrated to inhibit the proliferation and to induce the differentiation of various types of malignant cells, including breast, prostate and colon cancers as well as myeloid leukemia cells [14], [15]. The administration of 1,25(OH)_2_D_3_ and its analog has therapeutic effects in a mouse model of myeloid leukemia [16]. While ATRA induces granulocytic differentiation [6], [7], 1,25(OH)_2_D_3_ induces the differentiation of HL60 cells and other myeloid leukemia cells towards the monocyte and macrophage lineage [17], [18]. Interestingly, ATRA induces monocytic differentiation of monoblastic leukemia U937 and THP-1 cells [13], [19]. Combined treatment with 1,25(OH)_2_D_3_ and retinoids induces the differentiation of HL60 cells and human monoblastic leukemia cells, such as THP-1 cells, to monocyte/macrophage-lineage cells more effectively than 1,25(OH)_2_D_3_ alone [10], [12], [20]. Although 1,25(OH)_2_D_3_ has been shown to exert its biological effects on cellular proliferation and differentiation by genomic and/or non-genomic pathways [21], the detailed mechanisms remain unclear. Macrophages have been classified into two cell types, classically activated M1 macrophages and alternatively activated M2 macrophages [22], [23]. While M1 macrophages produce proinflammatory cytokines and enhance microbicidal and tumoricidal immunity, M2 macrophages are involved in wound healing and immune regulation. Although retinoids and 1,25(OH)_2_D_3_ play functional roles in monocytes and macrophages [4], [24], the macrophage cell type resulting from 1,25(OH)_2_D_3_ and/or retinoid differentiation of myeloid leukemia cells has not been further characterized. In this study, we examined the effects of 1,25(OH)_2_D_3_ in combination with retinoids on differentiation of myeloid leukemia cells and found that 1,25(OH)_2_D_3_ in combination with 9cRA and ATRA induce the differentiation of myeloid leukemia cells to macrophages with M2-like phenotype.

## Materials and Methods

### Compounds

1,25(OH)_2_D_3_, ATRA and 9cRA were purchased from Wako Pure Chemical Industries (Osaka, Japan). Am80 (4-[(5,6,7,8-tetrahydro-5,5,8,8-tetramethyl-2-naphthalenyl)carbamoyl]benzoic acid) [25] and HX630 (4-[2,3-(2,5-dimethyl-2,5-hexano)dibenzo[*b*,*f*][1], [4]-thiazepin-11-yl]benzoic acid) [26] were kindly provided by Dr. Koichi Shudo of Research Foundation ITSUU Laboratory (Tokyo, Japan).

### Cell culture, cell growth, nitroblue tetrazolium (NBT) reduction, and interleukin-10 (IL-10) production

Human myeloid leukemia HL60 and THP-1 cells (RIKEN Cell Bank, Tsukuba, Japan) were maintained in RPMI1640 medium containing 10% fetal bovine serum, 100 unit/ml penicillin, and 100 µg/ml streptomycin in a humidified atmosphere containing 5% CO_2_. Suspensions of cells (10^5^ cells/ml) were cultured with or without test compounds at pharmacological concentrations (30−100 nM) according to our preliminary experiments and the previous reports [10]–[12], [27]. Cell numbers were counted in a Z1S Coulter Counter (Beckman Coulter, Fullerton, CA). Cell morphology was examined in cell smears stained with May-Grünwald-Giemsa. NBT reduction was assayed colorimetrically and NBT-reducing activity data were normalized to the cell numbers [28]. IL-10 levels in culture media were determined with the Human IL-10 ELISA MAX Standard kit (BioLegend, San Diego, CA).

### Flow cytometry

Expression of cell surface antigens, CD14, CD11b and CD163, were determined with immunofluorescence staining and flow cytometry [29]. FITC mouse anti-human CD14, PE mouse anti-human CD11b, PE mouse anti-human CD163, and isotype control antibodies were purchased from Becton, Dickinson and Company (Franklin Lakes, NJ). The stained cells were assayed with a flow cytometer (BD FACSCalibur; Becton, Dickinson and Company) and analyzed with the BD CellQuest software (Becton, Dickinson and Company).

### Reverse transcription and real-time quantitative polymerase chain reaction

Total RNAs from samples were prepared by the acid guanidine thiocyanate-phenol/chloroform method [30]. cDNAs were synthesized using the ImProm-II Reverse Transcription system (Promega Corporation, Madison, WI). Intron-spanning primers were as follows: *CD163* (GenBank accession no. NM_004244), 5′-ACT GCA AGA ACT GGC AAT GG-3′ and 5′-CCA TGC TTC ACT TCA ACA CG-3′; *ARG1* (GenBank accession no. NM_000045), 5′-TCC AAG GTC TGT GGG AAA AG-3′ and 5′-ATT GCC AAA CTG TGG TCT CC-3′; *IL10* (GenBank accession no. NM_000572), 5′-CCA AGA CCC AGA CAT CAA GG-3′ and 5′-GGC CTT GCT CTT GTT TTC AC-3′; *IL12B* (GenBank accession no. NM_002187), 5′-ATT GAG GTC ATG GTG GAT GC-3′ and 5′-TTC TTG GGT GGG TCA GGT TT-3′; *TGFB1* (GenBank accession no. NM_000660), 5′-CAA CAA TTC CTG GCG ATA CCT C-3′ and 5′-AAA GCC CTC AAT TTC CCC TC-3′; *TNF* (GenBank accession no. NM_000594), 5′-TGC TTG TTC CTC AGC CTC TT-3′ and 5′-TGA GGT ACA GGC CCT CTG AT-3′; *IL6* (GenBank accession no. NM_000600), 5′-AAA GAG GCA CTG GCA GAA AA-3′ and 5′-AAA GCT GCG CAG AAT GAG AT-3′; *NOS2* (GenBank accession no. NM_000625), 5′-TAC CCC TCC AGA TGA GCT TC-3′ and 5′-TCT CCT TTG TTA CCG CTT CC-3′. Other primer sequences have been reported previously [28]. The mRNA values were normalized to the amount of β-actin mRNA.

### Statistical analysis

All values are shown as mean ± S.D. We performed one-way ANOVA followed by Tukey’s multiple comparisons or two-way ANOVA to assess significant differences using Prism 6 (Graphpad Software, La Jolla, CA).

## Results

### Induction of differentiation of human myeloid leukemia cells by retinoids plus 1,25(OH)_2_D_3_

**Figure 1:**
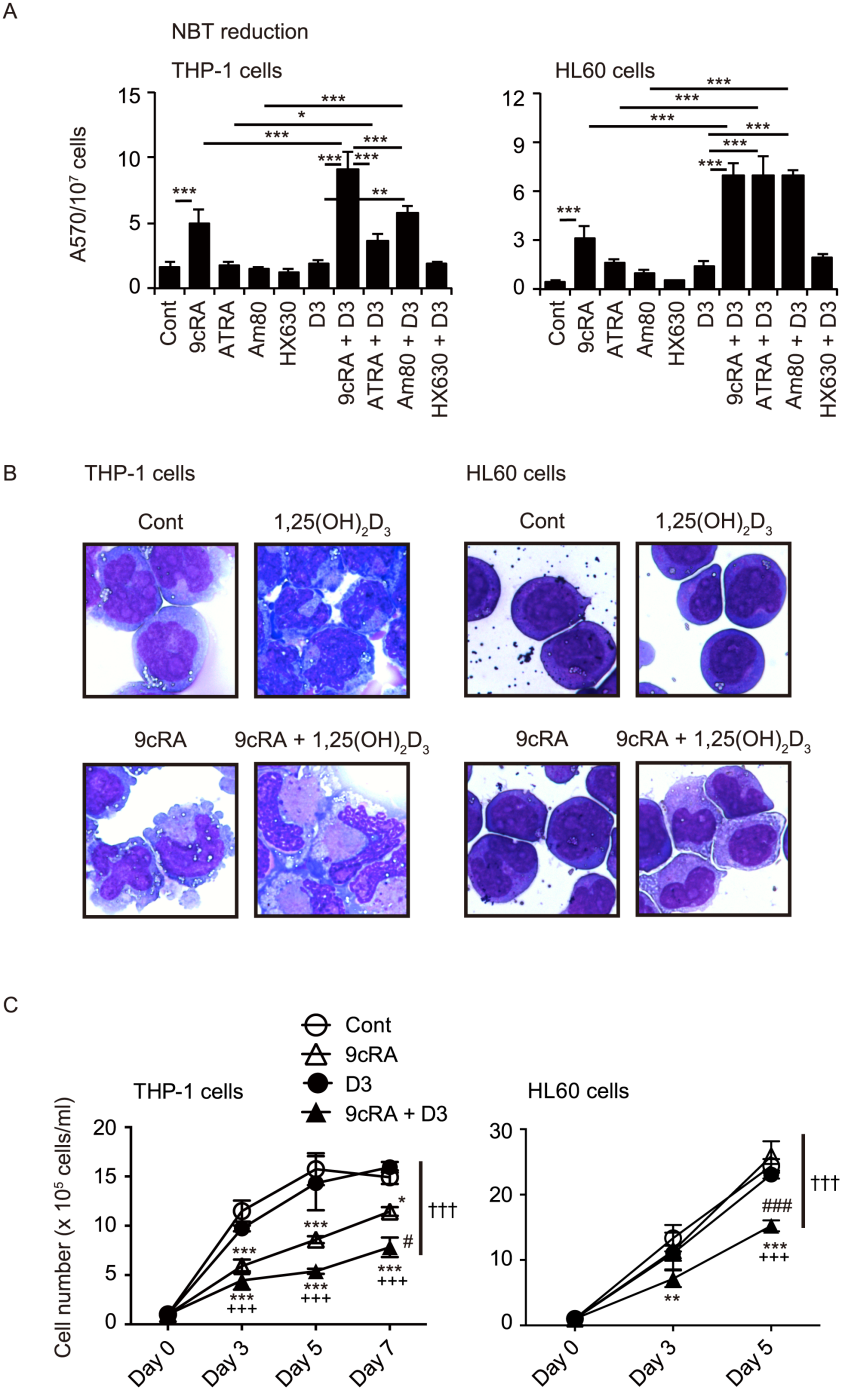
Induction of differentiation of THP-1 and HL60 cells by combined treatment with retinoid and 1,25(OH)_2_D_3_. (A) NBT-reducing activities. Cells were treated with vehicle control (Cont), 100 nM 9cRA, ATRA, Am80 or HX630 in the absence or presence of 100 nM 1,25(OH)_2_D_3_ (D3) for 5 days. *, *p*<0.05; **, *p*<0.01; ***, *p*<0.001 (one-way ANOVA followed by Tukey’s multiple comparisons). (B) Morphological changes of THP-1 and HL60 cells treated with 9cRA and/or 1,25(OH)_2_D_3_. Cells were treated with vehicle control (Cont), 100 nM 9cRA and/or 100 nM 1,25(OH)_2_D_3_ for 5 days and the cell smears were stained with May-Grünwald-Giemsa. (C) Cell proliferations. Cells (1×10^5^/ml) were cultured with vehicle control (Cont), 100 nM 9cRA and/or 100 nm 1,25(OH)_2_D_3_ (D3), and cell numbers were counted at indicated days. *, *p*<0.05; **, *p*<0.01; ***, *p*<0.001 vs Cont; ###, *p*<0.001 vs 9cRA; +++, *p*<0.001 vs D3 (one-way ANOVA followed by Tukey’s multiple comparisons). †††, *p*<0.001 (two-way ANOVA).

We examined the effects of 9cRA and ATRA in the absence or presence of 1,25(OH)_2_D_3_ on NBT-reducing activity, a marker of myeloid differentiation, in monoblastic leukemia THP-1 cells and promyelocytic leukemia HL60 cells. 9cRA (100 nM) increased NBT-reducing activity in THP-1 and HL60 cells, while ATRA (100 nM) was not effective (Fig. 1A), consistent with the previous reports showing that 9cRA is more potent than ATRA in inducing differentiation of leukemia cells [20], [31], [32]. In combination with 1,25(OH)_2_D_3_, 9cRA and ATRA effectively increased NBT-reducing activity in these cells (Fig. 1A). Am80 (also called tamibarotene) is a potent synthetic RAR agonist that is used in the treatment of recurrent APL in Japan [33], [34]. HX630 is an RXR selective agonist derived from LE135, an RAR antagonist [26]. HX630 does not exhibit RAR antagonistic activity but enhances the differentiation-inducing activity of Am80 in HL60 cells [33]. We also examined the effects of Am80 and HX630 in the absence or presence of 1,25(OH)_2_D_3_. Although Am80 and HX630 at 100 nM were not effective, the combination of 1,25(OH)_2_D_3_ with Am80 but not HX630 significantly increased NBT-reducing activity in THP-1 and HL60 cells (Fig. 1A).

THP-1 cells and HL60 cells were treated with 1,25(OH)_2_D_3_ and/or 9cRA and the morphological features were examined. While untreated THP-1 cells had a basophilic cytoplasm and large nuclei with several nucleoli, cells treated with 9cRA (100 nM) had grayish, enlarged cytoplasm and slightly lobulated nuclei (Fig. 1B). Although 1,25(OH)_2_D_3_ (100 nM) treatment did not induce an apparent morphological change, the combination of 9cRA and 1,25(OH)_2_D_3_ enlarged the grayish cytoplasm area more effectively than 9cRA alone. Untreated HL60 cells have promyelocytic features, although they are not derived from APL having t(15;17) [8]. While HL60 treated with 1,25(OH)_2_D_3_ (100 nM) had slightly less basophilic cytoplasm and decreased nuclear-cytoplasmic ratio, 9cRA (100 nM) induced differentiation of HL60 cells into myelocytic cells having slightly lobulated nuclei and a decreased nuclear-cytoplasmic ratio (Fig. 1B). Combined treatment with 9cRA and 1,25(OH)_2_D_3_ enhanced the monocytic features of HL60 cells with enlarged grayish cytoplasm and a further decrease in the nuclear-cytoplasmic ratio. These findings are consistent with the previous reports showing monocytic differentiation of myeloid leukemia cells by 1,25(OH)_2_D_3_ plus 9cRA or ATRA [32], [35]. 9cRA (100 nM) suppressed the proliferation of THP-1 cells, and although 1,25(OH)_2_D_3_ (100 nM) was not effective, combined treatment with 1,25(OH)_2_D_3_ enhanced the anti-proliferative effect of 9cRA (Fig. 1C). While treatment with 9cRA (100 nM) or 1,25(OH)_2_D_3_ (100 nM) alone was not effective, the combined treatment with 9cRA and 1,25(OH)_2_D_3_ effectively suppressed HL60 proliferation (Fig. 1C).

**Figure 2:**
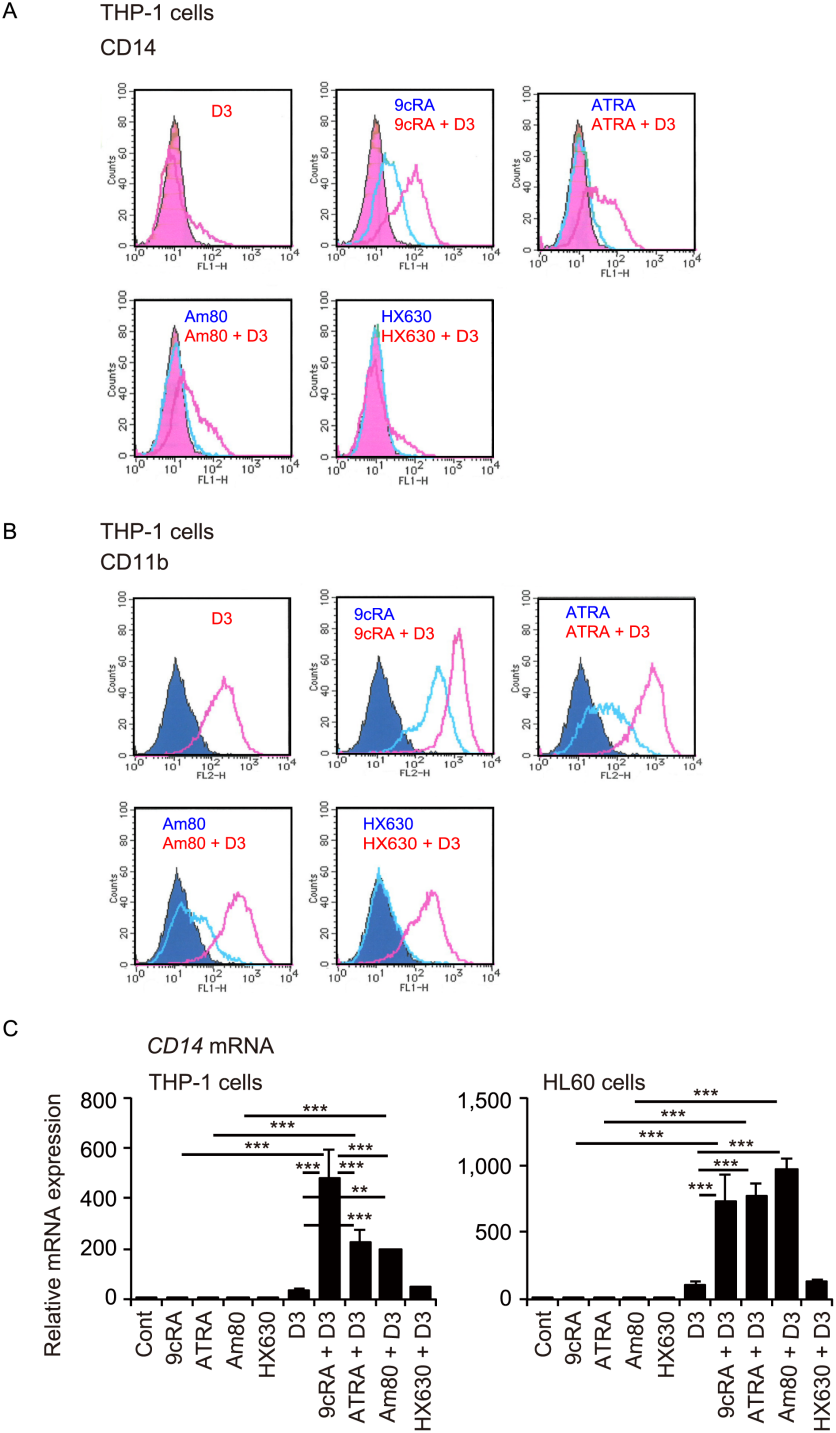
Effects of combined treatment with retinoid and 1,25(OH)_2_D_3_ on cell surface CD14 and CD11b expression and *CD14* mRNA expression. Representative histograms of CD14 expression (A) and CD11b expression (B) in THP-1 cells. Cells were treated with vehicle control (Cont), 100 nM 9cRA, ATRA, Am80 or HX630 in the absence or presence of 100 nM 1,25(OH)_2_D_3_ (D3) for 96 hours. Filled curves, vehicle control. Similar results were obtained from repeated experiments. (C) *CD14* mRNA levels in THP-1 and HL60 cells. Cells were treated with vehicle control (Cont), 30 nM 9cRA, ATRA, Am80 or HX630 in the absence or presence of 100 nM 1,25(OH)_2_D_3_ (D3) for 72 hours. **, *p*<0.01; ***, *p*<0.001 (one-way ANOVA followed by Tukey’s multiple comparisons).

We next examined the effects of combined treatment with retinoids and 1,25(OH)_2_D_3_ on expression of surface antigens, CD14 and CD11b, additional markers of myelomonocytic differentiation. 1,25(OH)_2_D_3_ at 100 nM slightly increased CD14 expression in THP-1 cells (Fig. 2A). 9cRA at 100 nM increased CD14 expression and the combination of 9cRA and 1,25(OH)_2_D_3_ increased CD14 expression more strongly than single use of these compounds. Interestingly, although ATRA and Am80 were not effective, these retinoids enhanced CD14 expression in combination with 1,25(OH)_2_D_3_. HX630 did not increase CD14 expression in the absence or presence of 1,25(OH)_2_D_3_. 1,25(OH)_2_D_3_ also slightly increased CD11b expression in THP-1 cells (Fig. 2B). 9cRA, ATRA and Am80 at 30 nM increased CD11b expression, and effectively enhanced CD11b expression induced by 1,25(OH)_2_D_3_. HX630 did not increase CD11b expression in the absence or presence of 1,25(OH)_2_D_3_. Transcriptional induction of *CD14*, a VDR target gene [36], is also associated with myeloid differentiation [28]. Retinoid treatment did not increase *CD14* mRNA levels in THP-1 and HL60 cells (Fig. 2C). Interestingly, while the effect of 1,25(OH)_2_D_3_ (100 nM) alone was not significant, combinations of 1,25(OH)_2_D_3_ with 9cRA, ATRA and Am80 effectively increased *CD14* mRNA expression in these cells. The combination of HX630 and 1,25(OH)_2_D_3_ was not effective in *CD14* mRNA induction. Thus, when combined with 1,25(OH)_2_D_3_, a RAR/RXR agonist (9cRA) and RAR agonists (ATRA and Am80), but not a RXR agonist (HX630), effectively induce differentiation of myeloid leukemia THP-1 and HL60 cells.

### Induction of M2 macrophage markers in human myeloid leukemia cells by retinoids plus 1,25(OH)_2_D_3_

**Figure 3:**
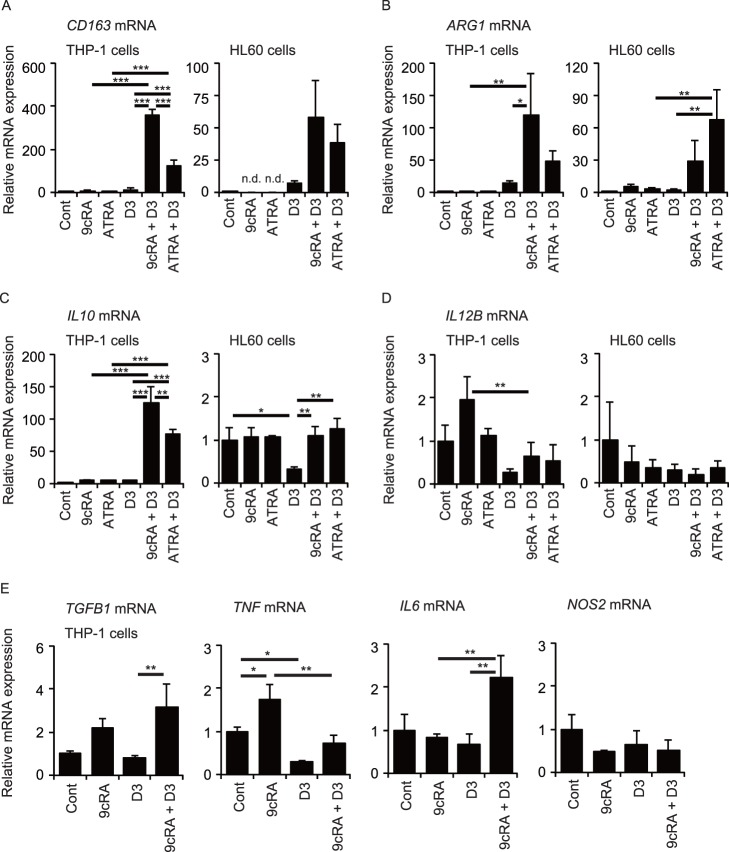
Expression of marker genes of M1 and M2 macrophages. mRNA levels of the M2 markers *CD163* (A), *ARG1* (B), *IL10* (C), and the M1 marker *IL12B* (D) in THP-1 and HL60 cells. (E) mRNA levels of the M2 marker *TGFB1*, the M1 markers *TNF*, *IL6*, and *NOS2* in THP-1 cells. Cells were treated with vehicle control (Cont), 30 nM 9cRA, or ATRA in the absence or presence of 100 nM 1,25(OH)_2_D_3_ (D3) for 72 hours. *, *p*<0.05; **, *p*<0.01; ***, *p*<0.001 (one-way ANOVA followed by Tukey’s multiple comparisons). n.d., not detected.

As shown in Figure 1B and the previous reports [10], [20], [32], combined treatment with retinoid and 1,25(OH)_2_D_3_ induces differentiation of myeloid leukemia cells into the monocytic lineage rather than the granulocytic lineage. We examined whether leukemia cells treated with retinoid plus 1,25(OH)_2_D_3_ exhibit M1 or M2 macrophage phenotypes. Expression of the *CD163*, *ARG1*, *IL10* and *TGFB1* marker genes is associated with M2 macrophage activation, whereas *IL12B*, *TNF*, *IL6* and *NOS2* expression is increased in M1 macrophages [23]. Treatment of THP-1 cells with 9cRA, ATRA or 1,25(OH)_2_D_3_ alone did not induce *CD163* mRNA expression, but the combination of 9cRA and 1,25(OH)_2_D_3_ effectively increased *CD163* mRNA levels (Fig. 3A). ATRA plus 1,25(OH)_2_D_3_ also increased *CD163* expression but less effectively than 9cRA plus 1,25(OH)_2_D_3_. Although *CD163* mRNA expression was not detected in HL60 cells treated with 9cRA or ATRA alone, 9cRA plus 1,25(OH)_2_D_3_ and ATRA plus 1,25(OH)_2_D_3_ tended to increase *CD163* mRNA levels. 9cRA plus 1,25(OH)_2_D_3_ also effectively increased *ARG1* mRNA expression in THP-1 cells, while ATRA plus 1,25(OH)_2_D_3_ tended to increase its expression, an effect that did not reach statistical significance (Fig. 3B). Interestingly, combination of ATRA and 1,25(OH)_2_D_3_ effectively increased *ARG1* mRNA levels in HL60 cells, while the combined effect of 9cRA and 1,25(OH)_2_D_3_ did not reach statistical significance. *IL10* mRNA expression was also elevated in THP-1 cells treated with 9cRA plus 1,25(OH)_2_D_3_ and, to a lesser extent, ATRA plus 1,25(OH)_2_D_3_ (Fig. 3C). Although treatment of HL60 cells with 1,25(OH)_2_D_3_ decreased *IL10* mRNA levels, the combination of 1,25(OH)_2_D_3_ with 9cRA or ATRA increased them to control levels. Combination of 1,25(OH)_2_D_3_ with 9cRA or ATRA did not increase *IL12B* mRNA levels in THP-1 and HL60 cells (Fig. 3D). *IL12B* mRNA levels in THP-1 cells treated with 9cRA were decreased by combined treatment with 1,25(OH)_2_D_3_. Addition of 9cRA increased *TGFB1* mRNA levels in THP-1 cells treated with 1,25(OH)_2_D_3_ (Fig. 3E). 9cRA increased *TNF* mRNA expression in THP-1 cells but combined treatment with 1,25(OH)_2_D_3_ decreased its expression. *IL6* mRNA expression was increased by the combination of 9cRA and 1,25(OH)_2_D_3_. 9cRA and/or 1,25(OH)_2_D_3_ induced no significant change in *NOS2* mRNA expression. Thus, the combination of retinoid and 1,25(OH)_2_D_3_ increases expression of genes associated with M2 macrophages.

**Figure 4:**
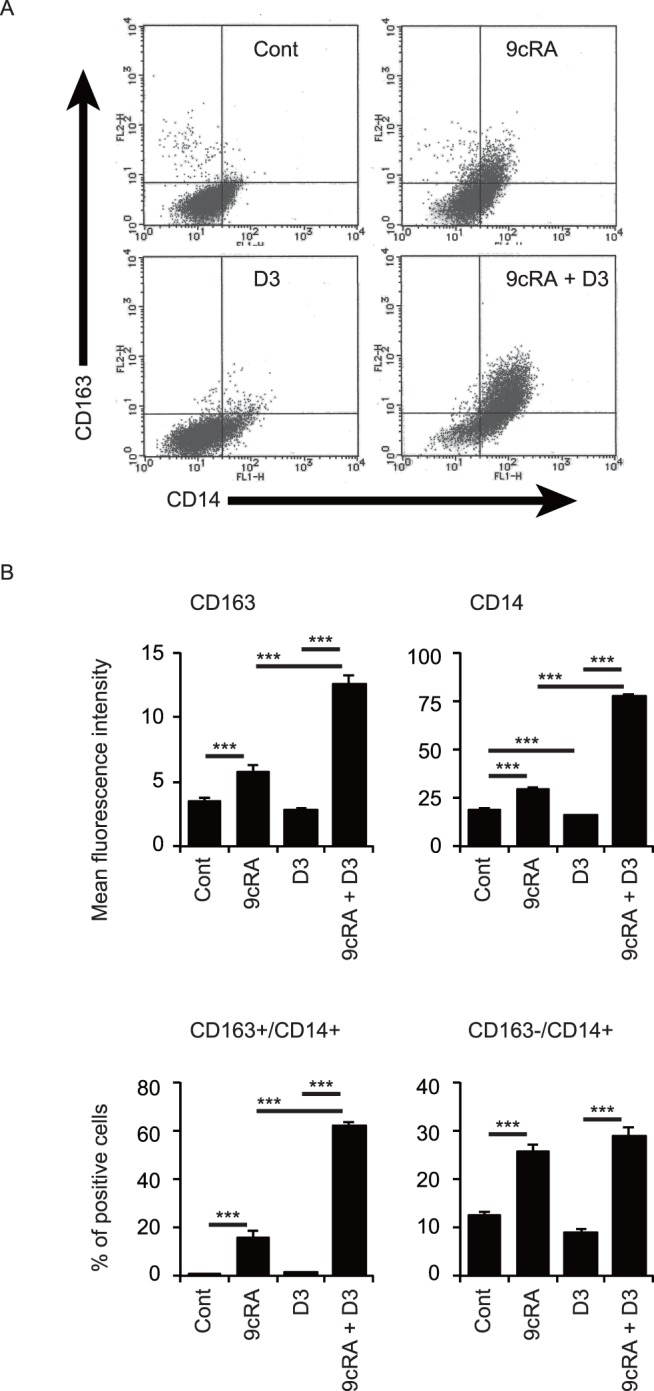
Cell surface expression of CD163 in THP-1 cells. (A) Representative histograms of CD14 and CD163 expression. (B) Quantification of mean fluorescence intensity of CD163 and CD14 expression. (C) Quantification of percentages of CD163+/CD14+ cells and CD163−/CD14+ cells. Cells were treated with vehicle control (Cont), 100 nM 9cRA and/or 100 nM 1,25(OH)_2_D_3_ (D3) for 96 hours. **, *p*<0.01; ***, *p*<0.001 (one-way ANOVA followed by Tukey’s multiple comparisons).

We further examined expression of CD163 as a cell surface marker of M2 macrophages in THP-1 cells. Figure 4A shows representative flow cytometric analysis of THP-1 cells treated with or without 9cRA and 1,25(OH)_2_D_3_ using anti-CD14 and anti-CD163 antibodies. 9cRA but not 1,25(OH)_2_D_3_ increased CD163 mean fluorescence intensity in THP-1 cells, and the combination of 9cRA and 1,25(OH)_2_D_3_ effectively enhanced the intensity values (Fig. 4B). 9cRA increased and 1,25(OH)_2_D_3_ slightly decreased surface CD14 mean fluorescence intensity, but 9cRA plus 1,25(OH)_2_D_3_ also strongly increased the CD14 intensity (Fig. 4B). 9cRA treatment increased the percentage of both CD163+/CD14+ cells and CD163−/CD14+ cells (Fig. 4B). Combination of 1,25(OH)_2_D_3_ with 9CRA increased the percentage of CD163+/CD14+ cells but not of CD163−/CD14+ cells. These findings are consistent with induction of the M2 macrophage phenotype in THP-1 cells by 9cRA plus 1,25(OH)_2_D_3_.

### The combination of 9cRA and 1,25(OH)2D3 induces IL-10 protein secretion in THP-1 and HL60 cells

**Figure 5:**
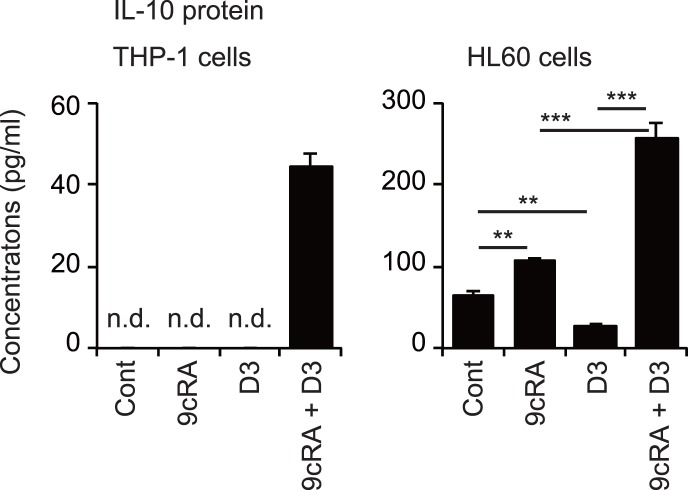
Secreted IL-10 production in THP-1 and HL60 cells. Cells were treated with vehicle control (Cont), 30 nM 9cRA and/or 100 nM 1,25(OH)_2_D_3_ (D3) for 72 hours and secreted IL-10 levels in media were measured. **, *p*<0.01; ***, *p*<0.001 (one-way ANOVA followed by Tukey’s multiple comparisons). n.d., not detected.

Finally, we examined IL-10 protein levels in conditioned media of THP-1 and HL60 cells treated with 9cRA and/or 1,25(OH)_2_D_3_. IL-10 protein levels from THP-1 cells treated with 9cRA and 1,25(OH)_2_D_3_ alone were below detection limits, but the combination of these compounds effectively induced IL-10 protein secretion from these cells (Fig. 5). While IL-10 protein was detected in the culture media of untreated HL60 cells, 9cRA increased and 1,25(OH)_2_D_3_ decreased the protein level. 9cRA plus 1,25(OH)_2_D_3_ effectively increased the IL-10 protein levels in media. Thus, combined treatment of leukemia cells with 9cRA and 1,25(OH)_2_D_3_ induces IL-10 protein secretion as well as increased expression of M2 macrophage markers.

## Discussion

In this study, we found that combined treatment with retinoid and 1,25(OH)_2_D_3_ induces the differentiation of human myeloid leukemia THP-1 and HL60 cells into the monocytic lineage with a M2 macrophage phenotype. ATRA induces granulocytic differentiation of promyelocytic leukemia HL60 cells but monocytic differentiation of monoblastic U937 and THP-1 cells [6], [7], [13], [19]. Combination of ATRA or 9cRA with 1,25(OH)_2_D_3_ effectively induces monocyte/macrophage phenotypes, such as phagocytic activity, monocyte-specific esterase, lysozyme secretion, and *CSF1R* expression, in HL60 and U937 cells [10], [32], [37]. We observed a monocytic morphology and increased CD14 expression in HL60 and THP-1 cells treated with 9cRA plus 1,25(OH)_2_D_3_ (Figs. 1 and 2). ATRA plus 1,25(OH)_2_D_3_ also induces differentiation of promyelocytic AML-193 cells into cells that display both a typical neutrophilic morphology and monocyte-specific properties, such as CD14 expression and monocyte-specific esterase, a hybrid granulomonocytic phenotype [35]. RAR signaling plays an important role in hematopoiesis and RARα is involved in neutrophil development [4]. RAR and RXR signaling pathways have been reported to regulate monocyte/macrophage function [4]. However, it remains to be determined how retinoid signaling enhances monocytic differentiation induced by 1,25(OH)_2_D_3_ in myeloid leukemia cells.

Among retinoids, 9cRA, ATRA and Am80, but not HX630, in combination with 1,25(OH)_2_D_3_ exhibit effective differentiation-inducing activity in these cells (Figs. 1 and 2). 1,25(OH)_2_D_3_ acts as a ligand for the nuclear receptor VDR, which forms a heterodimer with RXR [38], and the VDR−RXR heterodimer is not permissive to RXR ligand activation [39]. RAR selective ligands exhibit stronger synergistic effects with 1,25(OH)_2_D_3_ than RXR selective ligands in inhibiting proliferation and inducing differentiation of monoblastic U937 cells [27]. Combined effects of retinoid and 1,25(OH)_2_D_3_ on differentiation of myelomonocytic leukemia cells are likely mediated by VDR and RAR activation. RXR also forms heterodimers with RAR and other nuclear receptors, including peroxisome proliferator-activated receptor (PPAR) and liver X receptor (LXR) [38]. The RAR−RXR heterodimer is activated by RXR ligand only in the presence of RAR ligand, a feature known as conditional permissivity [39]. 9cRA exhibits differentiation-inducing activity more effectively than ATRA in the absence or presence of 1,25(OH)_2_D_3_ (Figs. 1 and 2), in agreement with previous reports [20], [31], [32]. Since 9cRA acts as a ligand for both RAR and RXR [40], synergistic activation may be due to binding to both RAR and RXR in the RAR−RXR heterodimer. In addition, RXR ligands can activate permissive heterodimers, such as PPAR−RXR and LXR−RXR [38]. PPARγ ligand and LXR ligand have been reported to induce differentiation of myeloid leukemia cells [41], [42]. RXR ligand activation of these permissive heterodimers may also contribute to the effect of 9cRA. However, the pure RXR ligand HX630 alone and in combination with 1,25(OH)_2_D_3_ was not effective in inducing differentiation of THP-1 and HL60 cells, while the combination of 1,25(OH)_2_D_3_ with the RAR selective agonist Am80 induced the differentiation of these cells (Figs. 1 and 2). These findings suggest that cooperation between VDR signaling and RAR signaling, not RXR signaling, plays a role in the differentiation of myeloid leukemia cells. VDR activation changes expression of many genes, including those involved in cellular proliferation, differentiation and apoptosis [21]. 1,25(OH)_2_D_3_ treatment can modulate intracellular kinase pathways via a non-genomic mechanism, and it remains unknown whether the non-genomic actions are mediated through VDR or other proteins [21]. Although both genomic and non-genomic effects of 1,25(OH)_2_D_3_ have been shown to play roles in differentiation induction of leukemia cells, the detailed mechanisms remain to be elucidated. RAR signaling may modulate the vitamin D signaling pathway or regulate other differentiation mechanisms. Further studies are needed to elucidate molecular mechanisms involving VDR, RAR and RXR signaling pathways in the induced differentiation of leukemia cells.

Combined treatment with 9cRA and 1,25(OH)_2_D_3_ increased mRNA expression of *CD163*, *ARG1*, *IL10*, and *TGFB1* genes (Fig. 3), surface expression of CD163 proteins (Fig. 4) and IL-10 secretion in THP-1 cells (Fig. 5). This combination also increased *CD163*, *ARG1* and *IL10* mRNA levels (Fig. 3) and IL-10 secretion in HL60 cells (Fig. 5). This phenotype has been characterized as M2 macrophages, although the classification of human macrophages remains controversial [23]. 9cRA plus 1,25(OH)_2_D_3_ did not increase expression of the M1 macrophage gene *IL12B* in THP-1 and HL60 cells (Fig. 3). Although *TNF* mRNA levels were not increased, *IL6* mRNA expression was effectively induced by the combination of 9cRA and 1,25(OH)_2_D_3_ in THP-1 cells. ATRA plus 1,25(OH)_2_D_3_ has been reported to induce mRNA and protein levels of tumor necrosis factor (TNF) and IL-6 in U937 cells [43] and to increase expression of inducible nitric oxide synthase (encoded by *NOS2*) and nitric acid production in U937 cells [44], while we observed no significant change in *NOS2* mRNA expression in THP-1 and HL60 cells after treatment with ATRA or 9cRA in combination with 1,25(OH)_2_D_3_ (Fig. 3, and data not shown). Tumor necrosis factor (TNF) and IL-6 are cytokines produced from M1 macrophages and *NOS2* expression is a M1 macrophage marker [22], [23], [45]. Macrophages with a mixed phenotype expressing both M1 and M2 markers have been identified [46]. Thus, differentiated leukemia cells by 9cRA plus 1,25(OH)_2_D_3_ are macrophage-like cells expressing primarily M2 markers with some M1 markers. Further analysis is required to reveal their functional characteristics.

The RAR signaling pathways play an important role in hematopoiesis and granulocytic differentiation [4], [47]. With regard to macrophages, ATRA inhibits TNF production in mouse peritoneal macrophages activated by lipopolysaccharide and interferon γ [48]. ATRA also reduces the synthesis of IL-12 and TNF and enhances IL-10 production in lipopolysaccharide-stimulated human macrophages [49]. Although VDR is dispensable for normal myelopoiesis [50], the vitamin D signaling pathway is involved in the regulation of macrophage/monocyte function [4]. 1,25(OH)_2_D_3_ suppresses activation of mouse macrophages by interferon γ [51], and enhances the immunoglobulin- and complement-dependent phagocytosis activity of human blood monocytes [52]. Thus, both ATRA and 1,25(OH)_2_D_3_ induce the macrophage/monocyte function common to M2 macrophages. Combined effects of retinoid and 1,25(OH)_2_D_3_ on physiological monocyte/macrophage function remain to be elucidated.

In contrast to 9cRA treatment, 1,25(OH)_2_D_3_ reduced *IL10* mRNA levels and IL-10 production in HL60 cells (Figs. 3 and 5). These findings agree with previous reports that show that 1,25(OH)_2_D_3_ suppresses *IL10* expression through VDR recruitment to the *IL10* promoter in monocytes [53], [54]. Interestingly, 1,25(OH)_2_D_3_ enhances *IL10* expression of activated human B lymphocytes by recruiting VDR to the *IL10* promoter [55]. The combination of 9cRA with 1,25(OH)_2_D_3_ effectively induced IL-10 transcription and secretion in THP-1 and HL60 cells (Figs. 3 and 5). Thus, VDR activation induces or suppresses *IL10* expression in a manner dependent on cellular conditions. Treatment with 9cRA plus 1,25(OH)_2_D_3_ increased *CD163* mRNA levels and surface CD163 expression (Figs. 3 and 4). CD163 mediates IL-10 secretion in human monocytes [56]. IL-10 plays an important role in immune regulation by macrophages [46], [57]. Our findings may provide an insight into mechanisms of IL-10 induction.

In conclusion, our results indicate that combined treatment with retinoid and 1,25(OH)_2_D_3_ induces differentiation of human myeloid leukemia THP-1 and HL60 cells into macrophage-like cells expressing M2 markers. Further study of human leukemia cell differentiation has the potential to extend differentiation-inducing therapy to the treatment of non-APL myeloid leukemia and to expand the understanding of human macrophage function.
